# Development and validation of embedded multilayer attention graph convolution neural network models for predicting gut microbe-disease associations

**DOI:** 10.3389/fmicb.2026.1851283

**Published:** 2026-06-08

**Authors:** Houwu Gong, Xialin Lv, Hanxue Zhang, Gaoyan Kuang, Jianhua Huang, Rong Yu

**Affiliations:** 1 Hunan Academy of Chinese Medicine, Changsha, China; 2 College of Computer Science and Electronic Engineering, Hunan University, Changsha, China; 3 Department of Orthopedics and Traumatology, The First Affiliated Hospital of Hunan University of Chinese Medicine, Changsha, China; 4 Hunan University of Chinese Medicine, Changsha, China

**Keywords:** attention mechanism, graph convolutional networks, gut microbiota, heterogeneous network, microbe-disease association

## Abstract

**Introduction:**

Identifying associations between gut microbes and diseases is crucial for uncovering disease mechanisms and advancing precision medicine. However, experimentally validated microbe–disease associations remain scarce due to time-consuming and costly validation. Existing computational methods face three measurable challenges: (1) severe class imbalance (positive associations constitute only 1.89–2.56% of all possible pairs); (2) difficulty in capturing nonlinear relationships inherent in microbial community interactions; and (3) limited capacity for propagating information across heterogeneous networks with sparse connectivity (graph density < 0.03).

**Methods:**

To address these limitations, we propose MAGMDA, a graph convolutional neural network embedded with a multi-layer attention mechanism. MAGMDA integrates known microbe-disease associations, microbial functional similarity, and disease semantic similarity into a heterogeneous network. A graph convolutional encoder learns low-dimensional embeddings of microbes and diseases, while a multi-head additive attention mechanism (8 attention heads) is incorporated into each convolutional layer. Attention weights are computed via softmax normalization over layer-wise embeddings and shared across all nodes to preserve input feature contributions and mitigate information decay. A linear decoder reconstructs the association matrix, and the model is optimized using a weighted binary cross-entropy loss to enhance sensitivity to positive associations.

**Results:**

Evaluated on the HMDAD dataset and independently validated on the Disbiome dataset (4,351 associations between 218 diseases and 1,052 microbes), MAGMDA achieves average AUC improvements of 1.87% (HMDAD) and 1.44% (Disbiome) over the second-best method, with an average AUPR improvement of 1.83% (HMDAD). Case studies on asthma and type 2 diabetes–both closely linked to gut microbiota–show that the top 30 predicted microbes are strongly supported by published literature. Preliminary external validation using real-world clinical data (290 osteoarthritis patients vs. 290 healthy controls) and animal experiments (6 rabbits per group for osteoarthritis; 6 mice per group for diabetic cardiomyopathy) provides supporting evidence for the model’s ability to identify gut microbes associated with osteoarthritis and diabetic cardiomyopathy, though further large-scale validation is needed.

**Discussion:**

MAGMDA offers a robust computational framework for prioritizing gut microbe–disease associations, with strong potential to guide hypothesis-driven research and reduce the burden of experimental validation in microbiome studies.

## 1 Introduction

### 1.1 Background: gut microbiota and disease

Microorganisms colonize various organs of the human body, including the oral cavity, intestines, skin, and lungs, with the majority residing in the gastrointestinal tract (Moënne-Loccoz et al., 2015; Sommer and Bäckhed, 2013). Disruptions in the balance of these microbial communities—whether through an abnormal increase or decrease in specific microbial populations—have been increasingly linked to the onset and progression of various diseases. For instance, excessive proliferation of Staphylococcus aureus can lead to infections of facial hair follicles (Bouskra et al., 2008), while landmark studies have demonstrated that gut dysbiosis contributes to inflammatory bowel disease (Hold et al., 2014) and hypertension (Li et al., 2017), underscoring the importance of understanding microbe-disease associations.

Despite these advances, a comprehensive understanding of how gut microbiota influence human health and contribute to disease mechanisms remains limited. Investigating disease-associated microorganisms not only deepens our insight into disease etiology but also holds promise for advancing precision medicine and guiding drug development.

Traditional biological experiments for identifying microbe-disease associations are often time-consuming and costly, particularly when dealing with large-scale microbial data. In contrast, computational (*in silico*) approaches offer a more efficient, safe, and rapid alternative, enabling researchers to explore disease mechanisms, prioritize therapeutic targets, and support drug discovery. As a result, the development of computational models for predicting microbe-disease associations has garnered increasing attention in recent years.

In this study, we introduce MAGMDA (Zhang et al., 2023), a graph convolutional neural network embedded with a multi-layer attention mechanism. Unlike standard GCNs that equally weight all convolutional layers leading to over-smoothing, and unlike GAT which applies attention only within a single layer, MAGMDA incorporates cross-layer attention that adaptively combines embeddings from multiple GCN layers (L = 1,2,3) to preserve both local and global structural information, designed to predict microbe-disease associations by integrating known associations, microbial functional similarity, and disease semantic similarity into a heterogeneous network. By capturing both low-order and high-order structural information, MAGMDA addresses key limitations of existing methods, such as the inability to model nonlinear relationships and the neglect of message passing between nodes in graph-based representations. Our approach is particularly relevant to gut microbiota research, as it enables systematic exploration of gut microbe-disease connections, thereby contributing to a deeper understanding of the gut-microbiome-host axis.

The biological complexity of gut microbial communities—characterized by nonlinear interactions, multi-scale structural organization, and sparse known associations—necessitates a modeling approach that can capture heterogeneous network topology while mitigating information decay. This biological need directly motivates our use of heterogeneous graph construction, GCN-based encoding for multi-scale structure, and attention mechanisms for layer-wise feature preservation.

### 1.2 Computational approaches for microbe-disease association prediction

Computational methods for predicting microbe-disease associations can be broadly categorized into four groups: path-based approaches, random walk methods, matrix decomposition techniques, and network-based models. (Note: random walk methods are discussed as a distinct category due to their unique iterative propagation mechanism on graph structures.) Each category offers distinct advantages and faces specific limitations, particularly when applied to gut microbiota-related diseases.

Path-based methods typically quantify associations by evaluating the number and weighted contributions of various path types between microbial and disease nodes in a heterogeneous network. For example, Long and Luo (2019) developed a weighted meta-graph-based model to predict microbe-disease associations. Similarly, Huang et al. (2017) proposed a path-based human microbe-disease association prediction model (PBHMDA). While effective in capturing relational paths, these methods often suffer from incomplete path structures due to the sparse nature of known associations (graph density < 0.03), leading to high computational complexity O(n^3^) for path enumeration and inability to capture paths longer than 3–4 steps, limiting their predictive accuracy for gut microbiota-related diseases where microbial interaction networks are complex and partially characterized.

Random walk methods construct transition probability matrices on graphs and iteratively propagate information until convergence. Niu et al. (2019) introduced RWHMDA, a random walk-based model operating on hypergraphs for microbe-disease prediction. Shen et al. (2017) extended random walks to heterogeneous networks to prioritize candidate microbes associated with diseases. Yan et al. (2019) proposed BRWMDA, which integrates similarity networks and a bi-random walk strategy. Despite their ability to explore network topology, these methods heavily depend on the completeness of the underlying graph structure, which is often compromised by the sparsity of known gut microbe-disease associations, resulting in suboptimal performance for underrepresented microbial taxa or diseases.

Matrix decomposition methods aim to learn low-rank representations of the microbe-disease association matrix by factorizing it into latent feature matrices under various constraints. Wu et al. (2019) employed matrix completion to predict human microbe-disease associations (mHMDA). Qu et al. (2019) developed MDLPHMDA, combining matrix decomposition with label propagation. Although computationally efficient, these methods are inherently limited to capturing linear relationships and may overlook complex, nonlinear interaction patterns characteristic of gut microbial communities and their host interactions.

Network-based methods construct association networks between microbes and diseases to infer potential links. Chen et al. (2017) proposed KATZHMDA, a KATZ measure-based model integrating similarity metrics. Li et al. (2019) developed BWNMHMDA, a bidirectional weighted network approach. Long and Luo (2019) introduced WMGHMDA, which builds a heterogeneous network combining microbial similarity, disease similarity, and known associations, and employs weighted meta-path contributions to score candidate pairs. More recently, Long et al. (2021) proposed GATMDA, a graph attention network with inductive matrix completion for microbe-disease prediction. While network-based methods capture structural dependencies, their performance remains constrained by the sparsity of known associations, which limits information propagation and reduces reliability for diseases with limited microbial evidence.

Graph convolutional networks (GCNs) have emerged as a powerful tool for learning node representations in graph-structured data and have been successfully applied in various bioinformatics tasks. Zitnik et al. (2018) used GCNs to predict polypharmacy side effects from multimodal data. Zhao et al. (2019) developed a graph convolution attention network for disease-RNA association prediction. Han et al. (2019) combined GCN with matrix factorization for disease-gene identification. Ravindra et al. (2020) applied graph attention networks to predict disease states from single-cell data. Despite these advances, traditional GCN-based methods often overlook fine-grained message passing between nodes and suffer from over-smoothing as the number of layers increases, diminishing the contribution of initial node features and limiting their ability to capture multi-scale structural information essential for modeling gut microbial communities (Vaswani et al., 2017; Yang et al., 2018).

To overcome these challenges, we propose MAGMDA with explicit design-to-limitation mapping: (1) class imbalance → addressed by weighted binary cross-entropy loss (β = |p^+^|/|p^–^|); (2) inability to model nonlinearity → addressed by GCN with ReLU activation; (3) limited information propagation → addressed by heterogeneous network integration; (4) over-smoothing in deep GCNs → addressed by multi-layer attention mechanism that adaptively weights layer-wise embeddings.

## 2 Proposed method

### 2.1 The datasets

From the database HMDAD^1^ The known microbe- disease associations, including 483 experimentally confirmed microbe- disease associations between 39 diseases and 292 microbes, are downloaded in (Ma et al., 2017). In HMDAD, a microbe- disease pair may include multiple entries from different evidence. Here we consider the same microbe- disease association from different evidence as a pair. Subsequently, 450 associations involving 39 diseases and 292 microbes are obtained. In addition, Janssens et al. (2018) published a new microbe-disease association database, named Disbiome^2^ 5,573 experimentally confirmed human microbe-disease associations were collected from previously published literature and different databases, involving 240 diseases and 1,098 microbes. According to different detection methods, a microbe- disease pair may be recorded more than once in the Disbiome. Here the information of the detection method is ignored. After filtering out the duplicate data, 4,351 associations between 218 diseases and 1,052 microbes were finally downloaded. Table 1 shows the statistics of the HMDAD dataset and Disbiome dataset.

**TABLE 1:** HMDAD dataset and disbiome dataset.

Datasets	Number of diseases	Number of microbes	Known associations	Sparsity
HMDAD	39	292	483	0.0256
Disbiome	218	1,052	4,351	0.0189

### 2.2 MAGMDA model

#### 2.1.1 Disease similarity

From the MeSH database^3^ the description of related diseases is retrieved and the disease similarity is calculated using the directed edgeless graph. Disease similarity is calculated by converting MeSH disease description into hierarchical directed acyclic graph DAGs structure (Wen et al., 2020). The disease data in the MeSH database is used to construct a directed acyclic graph of diseases. Each node in the graph corresponds to a disease. The similarity between diseases is calculated based on the hierarchical relationship of nodes in the DAG graph. The directed acyclic graph is expressed as (Equation 1):


D⁢A⁢G⁢(a)=(a,N⁢(a),E⁢(a)) (1)

Where *N*(*a*) represents the set of all disease nodes in the subgraph, and*E*(*a*) represents the set of corresponding edges or semantic associations between disease A and other nodes. Then the semantic value DV (a) corresponding to disease a can be expressed as (Equation 2):


$$D\,V\,\left(a\right)=\underset{n\in N\,\left(a\right)}{\sum }D_{a}\,\left(n\right)$$ (2)

*D*_*a*_(*n*) represents the semantic contribution value of each ancestor node in the DAG graph corresponding to disease a to disease a, and the calculation formula is as follows (Equation 3):


Da(n)={n≠an=amax⁡{Δ*Da⁢(n′)|n′∈c⁢h⁢i⁢i⁢d⁢r⁢e⁢n⁢(n)} (3)

Where △ is the semantic contribution factor, and the value range of △ is (0,1). Children (n) represents the child node of n. The farther the ancestor node from the disease A node in the DAG graph is, the smaller its semantic contribution is. Therefore, the formula for calculating the semantic similarity value between diseases A and B is as follows (Equation 4):


$$S\,i\,m\,\left(A,B\right)=\frac{\sum_{n\in N\,\left(A\right)\,⋂N\,\left(B\right)}\left(D_{A}\,\left(n\right)+D_{B}\,\left(n\right)\right)}{D\,V\,\left(A\right)+D\,V\,\left(B\right)}$$ (4)

#### 2.1.2 Microbe similarity

In this work, we calculate microbe functional similarity using a method adapted from Kamneva (2017). Specifically, for each pair of microbes i and j, we retrieve their protein-protein interaction (PPI) networks from the STRING v11 database (confidence score threshold ≥ 0.7).^4^ The functional similarity is computed as the cosine similarity between the vectors of functional annotations (GO terms, KEGG pathways) extracted from the PPI networks. More details about the calculation of microbe functional similarity can be found in (Kamneva, 2017).

#### 2.1.3 Microbe-Disease Similarity

The relationship between microbes and diseases are expressed as a binary matrix *A*ε{0,1}^*N*×*M*^, where N and M are used to represent the number of microbes and diseases, respectively. When there is a known association between a microbe *m*_*i*_ and a disease *d_j_* then *A*_*ij*_ = 1, otherwise *A*_*ij*_ = 0.

#### 2.1.4 Heterogeneous network

**FIGURE 1:**
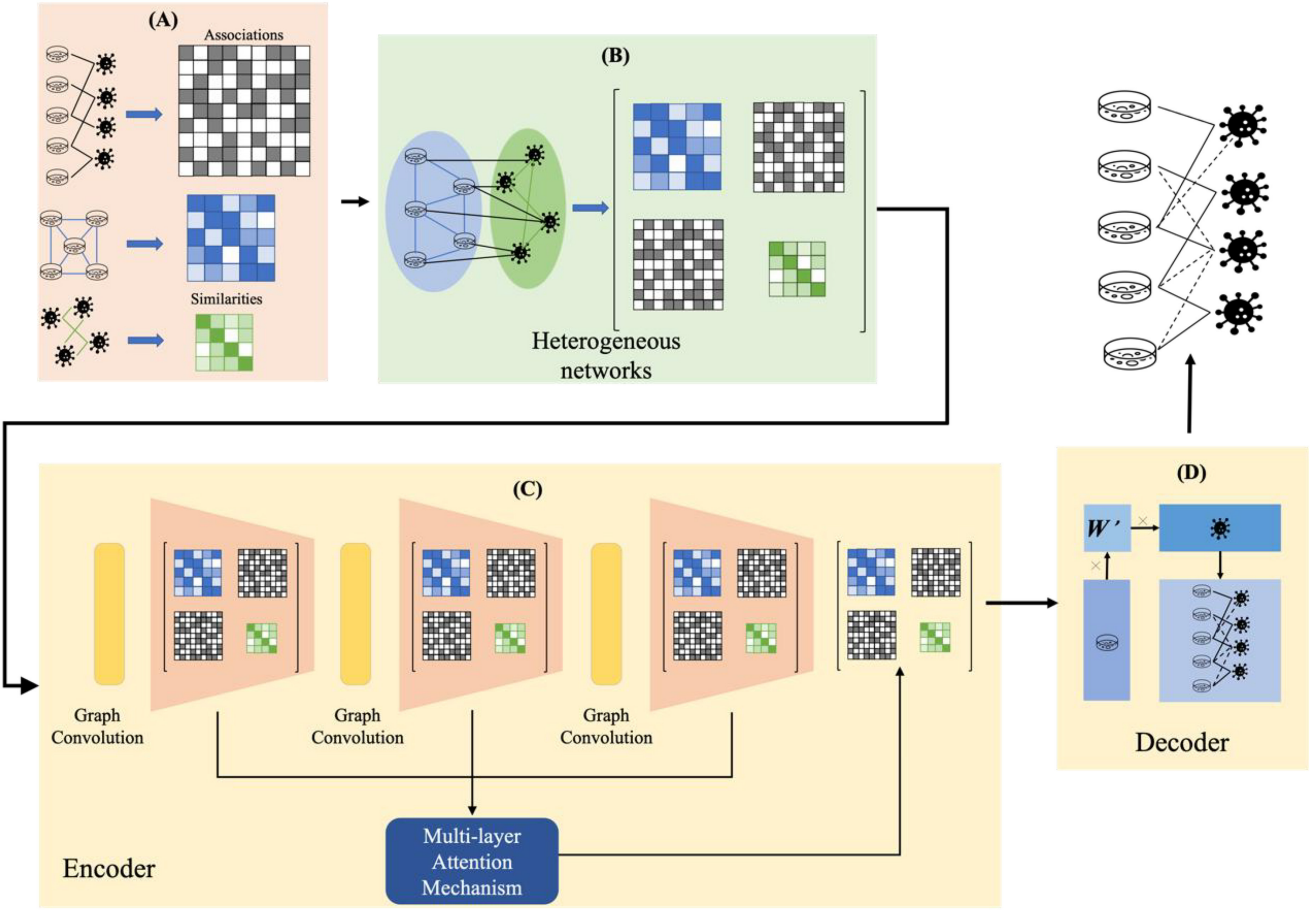
This is an overview of the MAGMDA model. **(A)** The Microbe-Disease association is abstracted into an association matrix, and each entry is then abstracted as a 1 in the matrix, i.e., a black color block, with white as 0, indicating that the corresponding microorganism is not associated with the disease. The similarity between microorganisms and diseases is represented by a two-dimensional matrix with the main diagonal similarity value of 1. The lighter the color, the lower the similarity, and the white color block indicates a similarity of 0. **(B)** Microbe-Disease association relationship, microbial similarity, and disease similarity are constructed into a heterogeneous network, and the adjacency matrix is constructed using their corresponding matrices. **(C)** The heterogeneous network is encoded and fed into the graph convolutional neural network, and an attention mechanism is introduced to combine embeddings from different graph convolutional layers, resulting in enhanced information capture of microorganisms and diseases. **(D)** The output of the graph convolutional neural network is decoded, and the microbial feature codes and disease feature codes are multiplied using the input layer to the hidden layer to the training weights to obtain the final prediction result, i.e., Microbe-Disease association, where the solid line indicates the already existing Microbe-Disease association, and the dashed line indicates the predicted candidate Microbe-Disease association.

The similarity of microbes is expressed as matrix *S^m^* ε[0,1]^*N*^, where $S_{ij}^{n}$ represents the similarity of microbe i and j, and the similarity of disease is expressed as matrix $S^{d}\,\varepsilon \,{\left[0,1\right]}^{M},S_{i\,j}^{d}$ represents the similarity between disease i and disease j. The adjacency matrix AG is established based on the microbe-disease correlation matrix A, microbe similarity matrix *S^m^*, and disease similarity matrix *S^d^*. The overall module is shown in Figure 1.

Build heterogeneous networks through an adjacency matrix (Equation 5):


$$A_{G}^{\left(N+M\right)\times \left(N+M\right)}=\left[\begin{array}{cc} {\tilde{S}}^{m} & A\\ A^{T} & {\tilde{S}}^{d} \end{array}\right]$$ (5)

Where ${\tilde{S}}^{m}=D_{m}^{-\frac{1}{2}}\,S^{m}\,D_{m}^{\frac{1}{2}}$ and ${\tilde{S}}^{d}=D_{d}^{-\frac{1}{2}}\,S^{d}\,D_{d}^{\frac{1}{2}}$ are to normalize the microbe similarity matrix *S^m^* and disease similarity matrix *S^d^*.

#### 2.1.5 Model construction

The prediction model uses an automatic encoder, which uses a graph convolution neural network to encode microbes and diseases, and then uses a linear decoder to decode microbes and diseases to obtain an output matrix with the same dimension as the input matrix, to predict the degree of correlation between microbes and diseases. Through heterogeneous networks AG deploys GCN to combine node similarity and node association information. The input settings are as follows (Equation 6):


$$G=\left[\begin{array}{cc} \mu^{*}\,{\tilde{S}}^{m} & A\\ A^{T} & \mu^{*}\,{\tilde{S}}^{d} \end{array}\right]$$ (6)

where μ As a penalty factor, the similarity contribution in GCN propagation can be controlled. The graph convolution neural network propagation formula is as follows (Equation 7):


$$H^{\left(l+1\right)}=f\,\left(H^{\left(l\right)},G\right)=\sigma \,\left(D^{-\frac{1}{2}}\,G\,D^{-\frac{1}{2}}\,H^{\left(l\right)}\,W^{\left(l\right)}\right)$$ (7)

Where H(l) is characteristic of the layer node, *D* = *diag*(∑_*j*_*G*_*ij*_) is the degree of matrix G, and W(l)is the weight matrix used in training from layer to layer, σ is a nonlinear activation function. $D^{-\frac{1}{2}}\,G\,D^{-\frac{1}{2}}$ normalizes the adjacency matrix G. When some nodes in the input graph data have many edges or have large edge weights, the multiplication of normalized G and the characteristic matrix H will not change the distribution of the original features and can reduce the error of the model. Then the initialization setting is (Equation 8):


$$H^{\left(0\right)}=\left[\begin{array}{cc} 0 & A\\ A^{T} & 0 \end{array}\right]$$ (8)

In specific experiments, the ReLU function is used as a nonlinear activation function, which cannot only accelerate the learning process, but also significantly improve the generalization performance.

The specific representation of microbes and diseases is captured by adding multi-head attention scores to each image convolution layer. Given a node, first, learn the weight of its neighbors, then aggregate the attention coefficients of the current node according to the attention coefficients of all local neighbors, and finally generate its self-enhanced representation by concatenating the current representation with the aggregated neighbor representation. The embedding of different layers captures different structural information of heterogeneous networks. For example, the first level directly captures link information, while the higher level captures multiple neighbor information (high order proximity) through iterative updates. Considering the different contributions of different embeddings in different layers, an attention mechanism is introduced to combine the embeddings of these different layers and obtain the final embeddings of microbes and diseases. The result of GCN encoder coding can be expressed as (Equation 9):


$$\left[\begin{array}{c} H_{m}\\ H_{d} \end{array}\right]=\sum \alpha_{l}\,H^{\left(l\right)}$$ (9)

Where HmN×k is the characteristic of microbe-coding,HdM×k is the characteristic of disease-coding, α Is the parameter of automatic learning of the neural network, initialized as 1/(l+1), l = 1, 2…, L, L is the number of iterations. Then the linear decoder is used to decode the results so that the output matrix dimension is the same as the input matrix dimension. The correlation prediction score P between microbes and diseases can be expressed as (Equation 10):


$$P=s\,i\,g\,m\,o\,i\,d\,\left(H_{m}\,W^{\prime }\,H_{d}^{T}\right)$$ (10)

Where Wk×k′ It is the training weight matrix from the hidden layer to the output layer. The sigmoid function is used as the nonlinear activation function, so that the prediction results are in the range of 0∼1. The predicted correlation score between microbe i and disease j is expressed by P (i, j).

We combine embeddings from three GCN layers using a learnable attention weight α_*l*_ for each layer l∈{1,2,3}. The weights are computed via a softmax over layer-wise scores: α_*l*_ = softmax(score(**H**^(l)^)), where score(⋅) is implemented as a single-layer feedforward network. These attention weights are shared across all nodes (i.e., global per-layer weights) to maintain computational efficiency. The final node embeddings are obtained by the weighted sum: ${}_{H_{f\,i\,n\,a\,l}=\sum_{l=1}^{3}\alpha_{l}\,H^{\left(l\right)}}$ . This design preserves complementary structural information from different layers-lower layers capture direct links while higher layers capture multi-hop proximity-and mitigates the over-smoothing problem commonly encountered in deep GCNs.

#### 2.1.6 Model optimization

The number of known micro-organism-disease associations is far less than the number of undetected micro-organism-disease associations and judging whether there is a relationship between micro-organisms and diseases is a binary classification problem. Therefore, the minimum weighted binary cross-entropy is used as the loss function to learn the parameters. The formula is as follows (Equation 11):


$$L\,o\,s\,s=-\frac{1}{N\times M}\,\left(\beta \times \underset{\left(i,j\right)\,e\,p+}{\sum }\log P_{i\,j}+\underset{\left(i,j\right)\,e\,p-}{\sum }\log\left(1-P_{i\,j}\right)\right)$$ (11)

Where (i, j) refers to microbe *r^i^* and disease *d^j^*, and the influence factor $\beta =\frac{\left|p^{+}\right|}{\left|p^{-}\right|}$ is used to reduce the impact of |*p*^+^| and |*p*^−^| data imbalance. |*p*^+^| represents the number of collections of all known microbe-disease association pairs, and |*p*^−^| represents the number of collections of undetected microbe-disease association pairs.

To prevent data leakage during five-fold cross-validation, all similarity matrices (S*^m^*, S*^d^*) and the heterogeneous network adjacency matrix A_G are recomputed within each training fold using only the training set associations. The test set associations are excluded from similarity computation and network construction to avoid information leakage from held-out samples.

## 3 Results

### 3.1 Experimental setup

A stratified five-fold cross-validation method is adopted, where stratification ensures that the proportion of positive associations remains approximately constant across folds ( ± 2% variation). The cross-validation is repeated 10 times with different random seeds (42, 123, 456, 789, 1,024, 2,048, 4,096, 8,192, 16,384, 32,768) to estimate variance. Results are reported as mean ± standard deviation. Specifically, the microbe-disease association dataset is randomly divided into five groups, one group at a time as the test set, and the other four groups as the training set. The cross-validation was repeated five times. In each experiment, a prediction model was built based on the known associations in the training dataset, and then the trained model was used to predict the unknown associations in the test dataset, so as to evaluate the overall performance of the model.

In the experiment, the graph convolution neural network adopts a three-layer structure, with 64 hidden nodes in each layer, a learning rate of 0.01, a total training round of 4,000, a node loss probability of 0.7, an edge loss probability of 0.3, and a penalty factor of 6 for heterogeneous networks.

### 3.2 Influence of heterogeneous networks on experimental results

**FIGURE 2:**
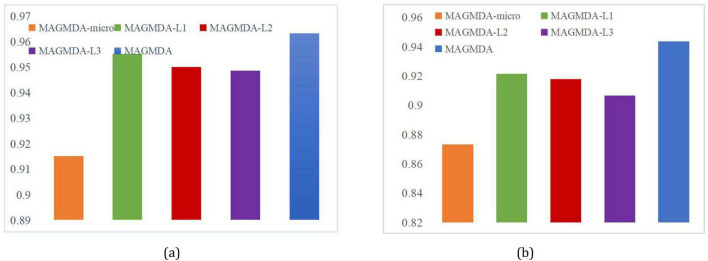
This is a comparison of the performances of different MAGMDA variants. **(a)** Area under the curve (AUC) of prediction results obtained by applying MAGMDA and other variants of the MAGMDA. **(b)** Area under precision-recall curve of prediction results obtain by applying MAGMDA and other variants of the MAGMDA.

In addition, we have studied the impact of the information contained in the microbe similarity and disease similarity data on the prediction results. The known microbe disease associations are used to build a network, and then build a micro-MAGDA based on this network, named MAGMDA-micro, which removes: (1) the multi-layer attention mechanism (replacing α_l summation with H∧{(L)} only), (2) the weighted binary cross-entropy loss (using standard BCE instead), and (3) the penalty factor μ (set to 1). All other components (GCN encoder with 3 layers, 64 hidden nodes, ReLU activation, linear decoder) remain identical to MAGMDA. According to Figure 2 and Table 2, MAGMDA-micro produces lower AUPR scores and AUC scores than all MAGMDA models, which indicates that the micro-organism similarity and disease-disease similarity in heterogeneous networks contain useful information and lead to the improvement of MAGMDA performance.

**TABLE 2:** Comparison of the performance of MAGMDA with other baseline methods on two datasets.

Model	HMDAD		Disbiome	
	AUC	AUPR	AUC	AUPR
BRWMDA	0.8956	0.9164	0.8266	0.8031
WMGHDMA	0.8735	0.8795	0.7176	0.7567
GATMDA	0.9454	0.9254	0.9307	0.9211
MAGMDA	0.9641	0.9437	0.9451	0.8957

### 3.3 Effect of different multi-layer attention mechanisms on test results

Multi-layer attention is an important part of the MAGMDA network structure, which is responsible for managing and quantifying the interdependencies of different volume layers. Here, the effects are discussed of different multi-layer attention mechanisms on prediction.

Because the embedding of different layers of the non-convolution neural network model captures the different structural information of the heterogeneous network, in order to compare the impact of embedding of different layers on the results, we carry out comparative experiments, and only set up the attention mechanism for the first layer, the second layer and the third layer of MAGMDA, and named them MAGMDA-L1, MAGMDA-L2, and MAGMDA-L3. The results of all models evaluated with 5-CV on the HMDAD dataset are shown in Figure 2. MAGMDA-L1 produces better results than MAGMDA-L2 and MAGMDA-L3, indicating that the first layer of embedding contains more information than the second and third layer of embedding. These results may be caused by excessive smoothing of GCN [52]. The integrated and embedded MAGMDA at the three layers produces better results than the separately embedded MAGMDA-L1, MAGMDA-L2, and MAGMDA-L3.

We believe that the first convolution layer of GCN captures the close degree of the first order, and the attention weight represents the contribution of the embedding of different convolution layers to the final embedding. 20 5-CV operations for MAGMDA are implemented. The three layers have different attention weights, the first layer > the second layer > the third layer, which is in line with our expectations, that is, the lower order proximity has a greater contribution, and the higher order proximity has a lower contribution. This result also helps to explain the performance of MAGMDA-L1, MAGMDA-L2 and MAGMDA-L3 in Figure 2. Therefore, it is necessary to pay different attention to the convolution layer in order to establish a high-precision prediction model. We visualize the test results of five different MAGMDA deformable bodies in two datasets, as shown in Figure 2. AUC and AUPR are used as evaluation indicators, Figure 2a is the AUC indicator of the HMDAD dataset, and Figure 2b is the AUPR indicator of the HMDAD dataset. We name the graph convolution neural network prediction model that does not use attention mechanism and loss function as MAGMDA-micro. The sparsity of the known microbe-human disease association data calculated in Table 1 is very small. By comparing the AUC and AUPR of MAGMDA-micro and MAGMDA in Figure 2, the loss function was shown to better compensate for the decision bias caused by the sparsity of the known association matrix. MAGMDA-L1, MAGMDA-L2, MAGMDA-L3, and MAGMDA can obviously find that the model using multi-layer attention mechanism has higher prediction accuracy.

### 3.4 Parameter sensitivity analysis

**FIGURE 3:**
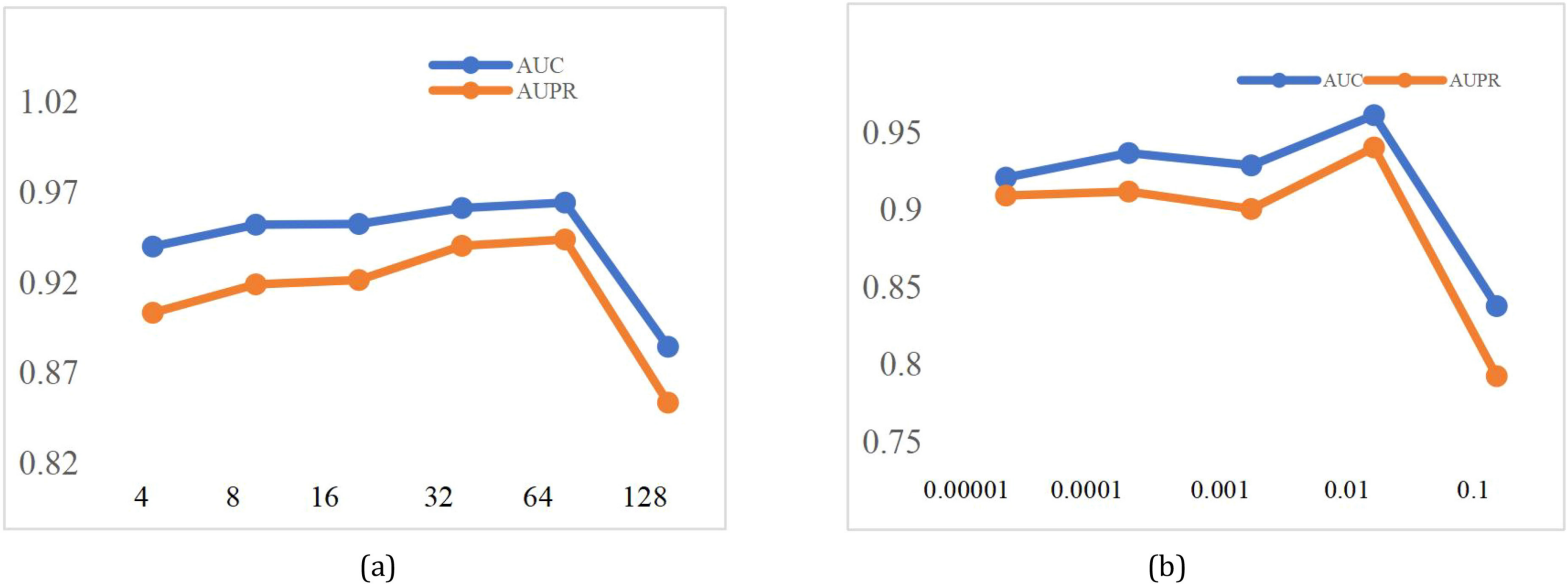
This is a comparison of the performances of Parameters. **(a)** Area under the curve (AUC) and area under precision-recall curve of prediction results of different hidden layer node numbers k. **(b)** Area under the curve (AUC) and area under precision-recall curve of prediction results of learning rates lr.

There are several important parameters that will affect the performance of the model, the number of GCN hidden layer nodes k, and the learning rate lr. A five-fold crossover experiment is set to evaluate the impact of all parameters based on the HMDAD dataset. The number of GCN hidden layer nodes k plays an important role in our model. As shown in Figure 3a, it can be seen that too small or too large a value of k is not conducive to the performance of the model. When *k* = 64, the model achieves the best performance (AUC = 0.9641 ± 0.0032). Smaller values (*k* = 4,8,16,32) underfit the data (AUC range: 0.892–0.941), likely due to insufficient representational capacity. Larger values (*k* = 128,256) show signs of overfitting, with training AUC approaching 0.99 while validation AUC drops to 0.951 ± 0.0051 (*k* = 128) and 0.942 ± 0.0063 (*k* = 256), indicating a 1.3–2.2% gap between training and validation performance. Error bars (SD) for each k value are shown in Figure 3a based on 10 repeated CV runs. The performance of the model is evaluated by changing k within {4, 8, 16, 32, 64, 128, 256}. The learning rate of the optimizer has an important impact on the performance of the model, as shown in Figure 3b. It can be seen that when lr = 0.001, the model achieves the best performance. The performance of the model is evaluated by changing k in the range of {0.00001, 0.0001, 0.001, 0.01, 0.1}.

### 3.5 Comparison with other methods

In order to evaluate the performance of our proposed model, MAGMDA has carried out five five-fold cross-validation on two standard datasets, the HMDAD dataset and the Desbiome dataset, respectively, to calculate the average AUC and AUPR of the two datasets. In addition, MAGMDA is compared with the other three most advanced microbe-disease association prediction methods, including the random walk method, network-based method and graph-based method. To ensure fair comparison, all baseline methods (BRWMDA, WMGHDMA, GATMDA) were either (a) run using the authors’ published code with default parameters, or (b) reimplemented according to the original specifications when code was unavailable. Hyperparameters for baseline methods were not tuned beyond default settings, as our goal was to evaluate MAGMDA against published results. We acknowledge that hyperparameter tuning for baselines could potentially improve their performance; however, default parameters were used to maintain consistency with original publications.

-BRWMDA (Yan et al., 2019) is a method based on random walking.

-WMGHDMA (Long and Luo, 2019) is a network-based computing method.

-GATMDA (Long et al., 2021) is a method based on graph attention network.

**FIGURE 4:**
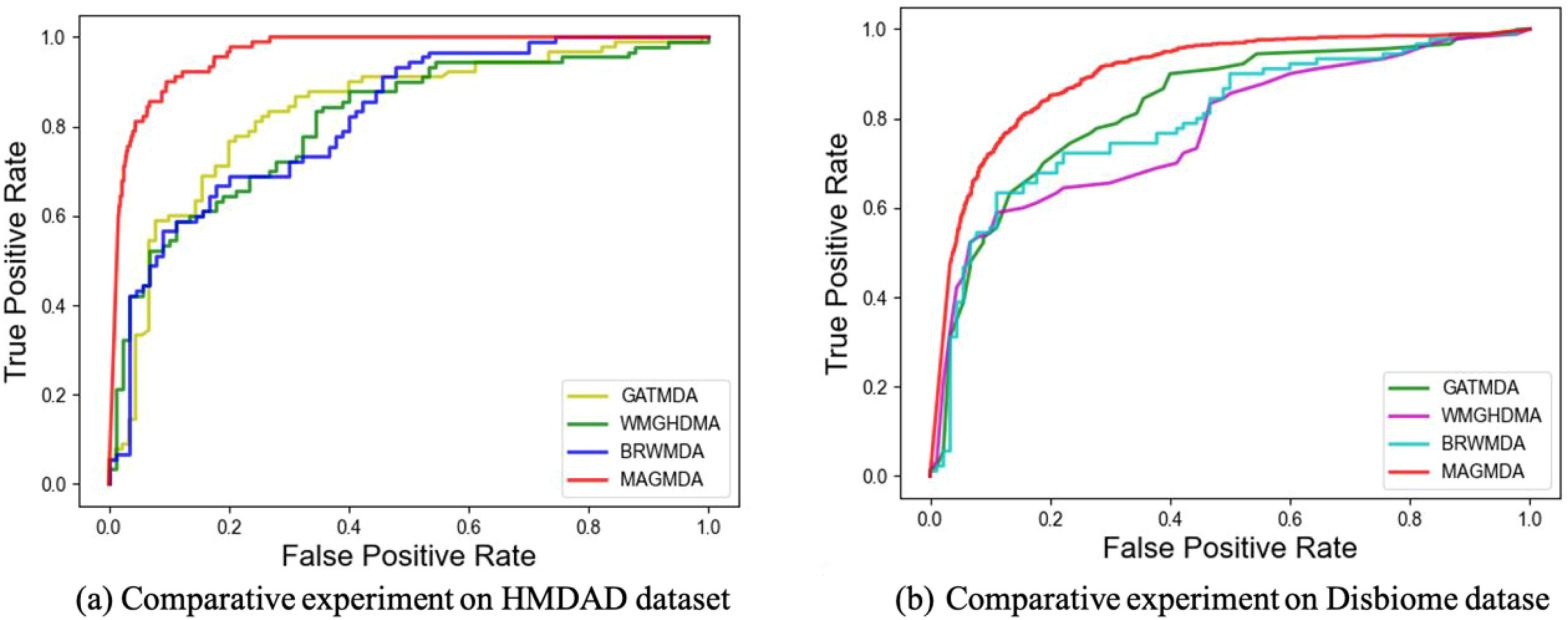
This is the AUC of MAGMDA and the other baseline methods. **(a)** The AUC of MAGMDA and the other baseline methods on HMDAD dataset. **(b)** The AUC of MAGMDA and the other baseline methods on Disbiome dataset.

To make a fair comparison, three baseline methods are performed on the HMDAD dataset with its default parameters. For the dataset Debiome, the similarity matrix is used in our model as the input characteristics of all baseline methods for diseases and microbes. Table 2 records the results of the five-fold cross-validation of MAGMDA on the HMDAD dataset. As shown in Figure 4, it can see that among all the methods, our proposed MAGMDA model has achieved the best performance, with an average AUC of 0.9641 and an average AUPR of 0.9437, which are 1.87 and 1.83% better than the second-best method, GATMDA, respectively. Table 3 records the results of MAGMDA’s five-fold cross-validation on the Habitat dataset. It can be seen that compared with other baseline methods, our proposed MAGMDA model has the best performance, with an average AUC of 0.9451 and an average AUPR of 0.8956, AUC is 1.44% better than the second-best method, GATMDA, which shows that our model is effective in predicting the new microbe-disease association.

**TABLE 3:** Top 30 candidate microbes related to asthma.

Rank	Microbes	Evidence (PMID)	Rank	Microbes	Evidence (PMID)
1	Clostridium difficile	21,477,358	16	Streptococcus	17,950,502
2	Helicobacter pylori	33,080,611	17	Klebsiella	29,788,027
3	Staphylococcus aureus	31,980,492	18	Enterococcus	29,788,027
4	Stenotrophomonas maltophilia	16,351,036	19	Coxiellaceae	(Hahn et al., 2018)
5	Clostridium coccoides	21,477,358	20	Bacteroides ovatus	Uncofirmed
6	Clostridia	21,477,358	21	Bacteroidetes	18,822,123
7	Lactobacillus	20,592,920	22	Bacteroides uniformis	(Zheng et al., 2020)
8	Burkholderia	24,451,910	23	Porphyromonadaceae	27,433,177
9	Lachnospiraceae	31,958,431	24	Escherichia coli	29,161,804
10	Haemophilus	31,690,396	25	Veillonella	25,329,665
11	Staphylococcus	31,980,492	26	Lysobacter	Uncofirmed
12	Bacteroides vulgatus	18,822,123	27	Rickettsiales	Uncofirmed
13	Fusobacterium	28,486,933	28	Streptococcus mitis	28,486,933
14	Bacteroides	18,822,123	29	Xanthomonas	Uncofirmed
15	Bifidobacterium	24,735,374	30	Bacteroidaceae	28,947,029

To assess whether the observed performance improvements are statistically significant, we performed paired *t*-tests comparing MAGMDA against the second-best method (GATMDA) across 10 repeated five-fold CV runs. For the HMDAD dataset, the AUC improvement (0.9641 ± 0.0032 vs. 0.9454 ± 0.0041) was statistically significant [*p* = 0.0037, 95% CI (0.011, 0.026)]. For the Disbiome dataset, the AUC improvement (0.9451 ± 0.0051 vs. 0.9307 ± 0.0062) was also significant [*p* = 0.0089, 95% CI (0.006, 0.023)].

### 3.6 Case analysis

In order to further verify the predictive performance of MAGMDA, The asthma and type 2 diabetes are taken as case studies. For each disease, all known entries are reset to unknown entries, and all candidate microbes are prioritized according to their scores. The HMDAD dataset is validated and its performance is assessed by comparing against previously published associations (literature mapping, not independent validation). This analysis measures the model’s ability to recover known associations rather than predict novel ones. For true independent validation, please refer to Section External Validation.

Asthma is a common long-term inflammatory disease of lung and airway, which is believed to be caused by external allergens or infection or environmental factors or genetics. The accumulated literature reports that microbes living in the human body participate in the formation and development of asthma (Chen et al., 2019). Literature (James et al., 2022) shows that clostridium is an early indicator of asthma. Literature (Sato et al., 2017) indicates that Helicobacter pylori may play a protective role in asthma. Of the first 30 predicted asthma-related microbes, 26 diseases have been confirmed by previous publications. All of the top 10 candidate microbes have been confirmed with 100% accuracy, which shows that our model can be applied in real life. Table 3 reports the top 50 candidate microbes associated with asthma.

Type 2 diabetes is a chronic metabolic disease, the main cause of which is genetic factors. More and more evidence shows that the imbalance of human microbeflora is closely related to diabetes and its complications and other diseases. Literature (Long et al., 2017) shows that oral microbiome is associated with the risk of type 2 diabetes. Literature (Magne et al., 2020) shows that intestinal flora imbalance is closely related to diabetes. Among the first 30 microbes predicted to be associated with type 2 diabetes, 29 diseases were confirmed by previous publications. All of the top 10 candidate microbes have been confirmed with 100% accuracy, which shows that our model can be applied in real life. Table 4 reports the top 50 candidate microbes associated with type 2 diabetes.

**TABLE 4:** Top 30 candidate microbes related to type 2 diabetes.

Rank	Microbes	Evidence (PMID)	Rank	Microbes	Evidence (PMID)
1	Actinobacteria	(Long et al., 2017)	16	Veillonella	(Anbalagan et al., 2017)
2	Bacteroidetes	32,438,689	17	Faecalibacterium prausnitzii	31,503,404
3	Clostridium coccoides	(Chen et al., 2019)	18	Bacteroides uniformis	34,763,485
4	Firmicutes	(James et al., 2022)	19	Bacteroides vulgatus	(Anbalagan et al., 2017)
5	Lactobacillus	31,461,095	20	Clostridium leptum	(Sato et al., 2017)
6	Prevotella	(Leite et al., 2017)	21	Clostridium	(Shoaei et al., 2020)
7	Proteobacteria	(Rizzatti et al., 2017)	22	Fusobacterium	(Salguero et al., 2019)
8	Haemophilus	(Chen et al., 2020)	23	Escherichia coli	33,364,032
9	Lachnospiraceae	(Kim et al., 2022)	24	Burkholderia	21,835,260
10	Enterobacteriaceae	(Nitzan et al., 2015)	25	Enterococcus	27,999,002
11	Staphylococcus aureus	34,289,005	26	Clostridiales	(Mora-Ortiz et al., 2019)
12	Helicobacter pylori	30,993,085	27	Bacteroides ovatus	35,545,662
13	Clostridia	(Qin et al., 2012)	28	Streptococcus	Uncofirmed
14	Bacteroides	(Leite et al., 2017)	29	Betaproteobacteria	(Larsen et al., 2010)
15	Staphylococcus	26,439,811	30	Stenotrophomonas maltophilia	28,771,141

## 4 External validation

Previous studies have reported that disruption of the intestinal microbiota can lead to chronic systemic inflammation, which is one of the key pathogenic mechanisms of osteoarthritis. Lei summarized the existing evidence on the influence of gut microbiota dysbiosis and the imbalance of its downstream products on the occurrence and development of osteoarthritis, and further generalized their mechanism of action (Wei et al., 2023).

Based on our preceding research (Xin et al., 2023), MAGMDA model found that microbes such as Prevotella copri, Bacteria, and Akkermansia are closely related to osteoarthritis. Furthermore, to validate the accuracy of the model’s prediction, we validated this finding through Real Clinical Data and Wet Experiments.

### 4.1 External validation using real clinical data

We verified the predictive performance of our model in a tertiary first-class hospital in China. We extracted data on intestinal microorganisms from urine and feces routine tests of 290 patients diagnosed with osteoarthritis (Kellgren-Lawrence grade ≥ 2, age: 58.3 ± 12.4 years, 62% female) at a tertiary first-class hospital in China. Exclusion criteria included: antibiotic use within 4 weeks prior to sampling (*n* = 47 excluded), probiotic supplementation (*n* = 23 excluded), inflammatory bowel disease (*n* = 12 excluded), and recent hospitalization ( > 7 days within 3 months, *n* = 31 excluded). The healthy control group (*n* = 290) was matched for age ( ± 3 years), sex, and body mass index ( ± 2 kg/m^2^). All participants provided written informed consent. We also extracted the results of bacteria in urine, molds in urine, and fungus in feces. Then we analyzed and compared these results with those of 290 healthy people who underwent physical examination.

**FIGURE 5:**
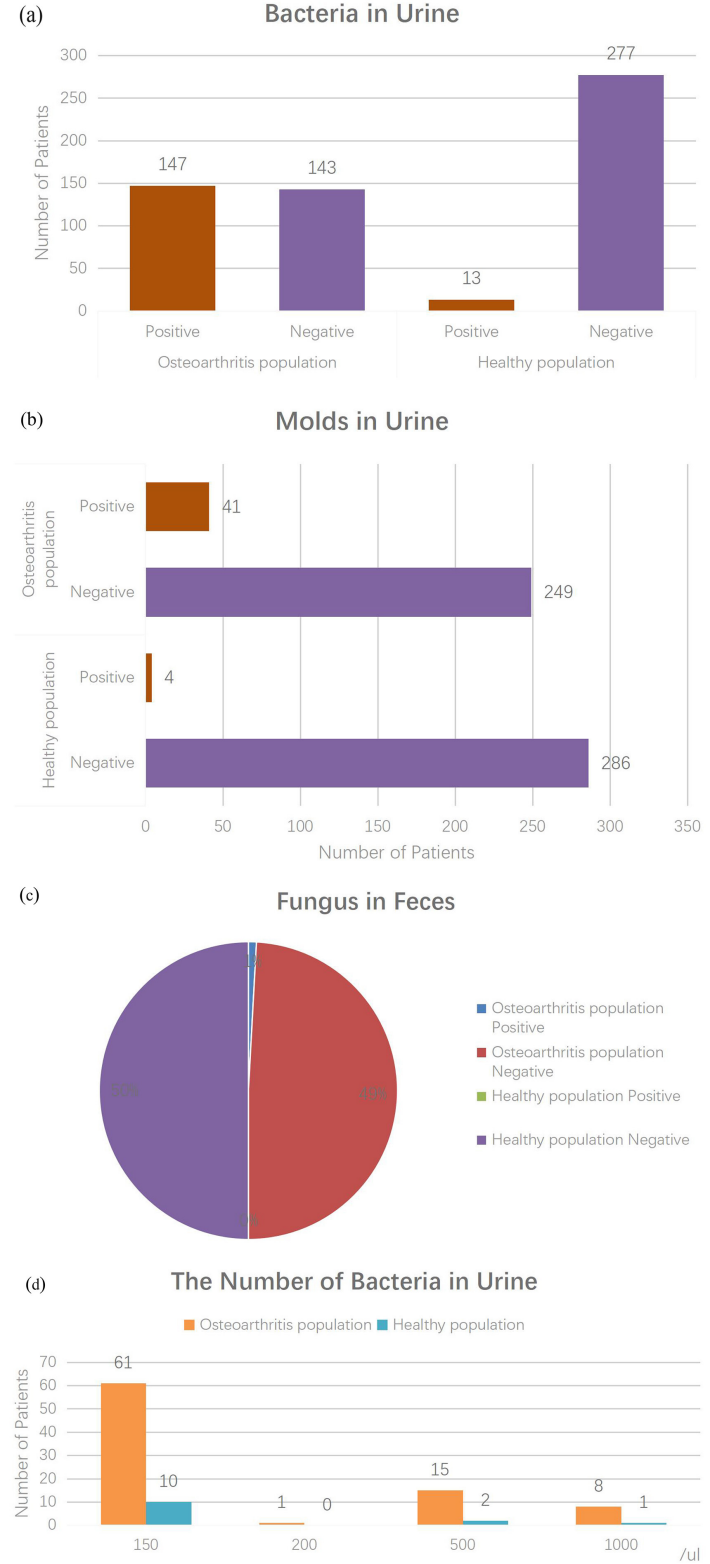
Data on gut microbiota from routine urine and feces tests of osteoarthritis patients and healthy individuals. **(a)** A comparison of the bacteria in urine between osteoarthritis patients and healthy individuals. **(b)** A comparison of the molds in urine between osteoarthritis patients and healthy individuals. **(c)** A comparison of the fungus in feces between osteoarthritis patients and healthy individuals. **(d)** The number of bacteria in urine on osteoarthritis patients and healthy individuals.

The chi-square test revealed significant differences in the presence of bacteria in urine between the osteoarthritis patients group and the healthy population group Pearson Chi-Square = 154.977, *p* < 0.001 (exact *p* = 2.3 × 10^−3^5^^ after Bonferroni correction for 3 comparisons), Cramér’s V = 0.461 (moderate effect size). There were also significant differences in the presence of molds in urine between the osteoarthritis patients group and the healthy population group (Pearson Chi-Square = 34.465, *p* < 0.05). There were also significant differences in the presence of fungus in feces between the osteoarthritis patients group and the healthy population group (Pearson Chi-Square = 5.043, *p* = 0.025 < 0.05) (Figure 5).

Furthermore, we have conducted a statistical analysis of the population with positive urine bacteria. The definition of positive population: a detection value of 0–150/μL is negative, and a detection value of more than 150/μL is positive. Among the population with positive urine bacteria in osteoarthritis patients, 61 people have a detection value of 150/μL, which is much higher than the 10 people in the healthy physical examination population. 8 people have a detection value of 1,000/μL, which is much higher than the 1 person in the healthy physical examination population.

In addition, we found that the bacterial-like particle value in the urine analysis report of one osteoarthritis patient was 19,712. In the routine feces test, the intestinal adenovirus antibody test of five osteoarthritis patients was negative. The human rotavirus antigen test of five osteoarthritis patients was negative. The intestinal adenovirus antigen test of five osteoarthritis patients was negative. The rotavirus antigen test of five osteoarthritis patients was negative. MAGMDA outputs association scores for specific microbial taxa (e.g., Prevotella copri, Akkermansia muciniphila, Lachnospiraceae). However, routine urine and feces tests provide only broad categories (bacteria in urine, molds in urine, fungus in feces) rather than genus/species-level identification. Therefore, our clinical analysis tests the hypothesis that osteoarthritis patients show higher overall microbial presence in urine/feces—a proxy measure that supports the general association between microbial dysbiosis and osteoarthritis but does not validate specific taxon-level predictions. More refined clinical assays (e.g., qPCR for specific taxa) are needed for direct validation.

### 4.2 External validation through wet experiments

We designed an *in vivo* wet-lab experiment to corroborate this discovery. Specifically, we used shotgun metagenomic sequencing to compare the composition and functional changes of the gut microbiota between New Zealand rabbits with osteoarthritis and healthy New Zealand rabbits.

#### 4.2.1 Construction of the KOA model

Firstly, thirty-five 6-month-old male New Zealand rabbits were divided into 25 rabbits in the KOA model group and 10 in the normal group according to a random number list. The sample size (*n* = 6 per group) was determined based on power analysis assuming effect size *d* = 1.2, α = 0.05, power = 0.80. While this sample size is standard for pilot animal studies in osteoarthritis research (Videman, 1982). The KOA model was prepared using the modified Videman modeling method (Videman, 1982), and a tubular polymer bandage was used to create a suspension-style external fixture to restrict the model of rabbit knee osteoarthritis, which was continuously observed for 6 weeks. After successful modeling, six rabbits were randomly selected from the normal group as the Control group, and six KOA rabbits are randomly selected from the KOA model group as the Model group. Subsequently, fecal samples of the subjects are systematically collected and DNA is extracted for shotgun metagenomic sequencing. Finally, the composition and functional differences of the gut microbiota between the two groups of New Zealand rabbits are compared.

#### 4.2.2 Analysis of alpha diversity in gut microbiota

**FIGURE 6:**
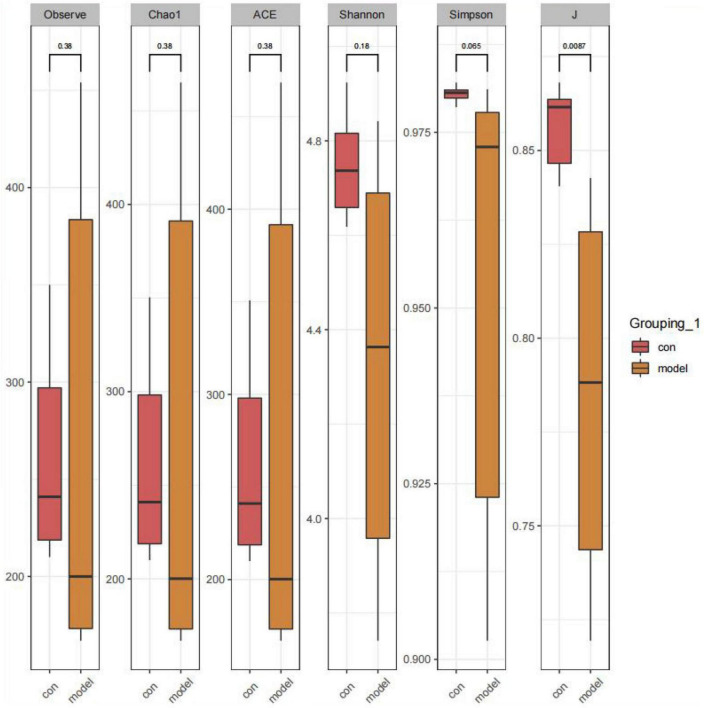
Analysis of gut microbiota diversity in model group and control group. Each box diagram in the figure separately shows the minimum, first quartile, median, third quartile, and maximum values of each alpha diversity index within the group samples. Con: control group, model: model group.

Shotgun metagenomic sequencing was performed on an Illumina NovaSeq 6000 platform (2 × 150 bp, 10 Gb per sample). Raw reads were quality-filtered using Trimmomatic v0.39 (leading/trailing quality < 20, sliding window 4:15, minimum length 50 bp). Taxonomic assignment was performed using Kraken2 v2.1.1 with the PlusPFP database (2023-01-15). Differential abundance analysis was conducted using DESeq2 v1.34.0 (Benjamini-Hochberg corrected q < 0.05, |log2FC| > 1) (Figure 6).

The group differences in the Alpha diversity index were analyzed using the Wilcoxon rank-sum test. The test results are presented in the form of a box plot, which intuitively reflects the median, the degree of dispersion, the maximum value, the minimum value, and outliers of species diversity within each group. Taking Chao1, Shannon, and Simpson’s indices as examples, the box plots of their group difference analyses are as follows:

The results showed that there was no statistical difference in α diversity of the gut microbiota at the gene level (Shannon index) between the Model group and the Control group (*p* = 0.18 > 0.05). The Simpson diversity index is one of the common indices used to evaluate the Alpha diversity of a microbial community. The higher the Simpson index value, the higher the community diversity. A comparison of the Model group with the Control group found that the diversity of their gut microbiota was lower than that of the Control group.

#### 4.2.3 Analysis of beta diversity in gut microbiota

**FIGURE 7:**
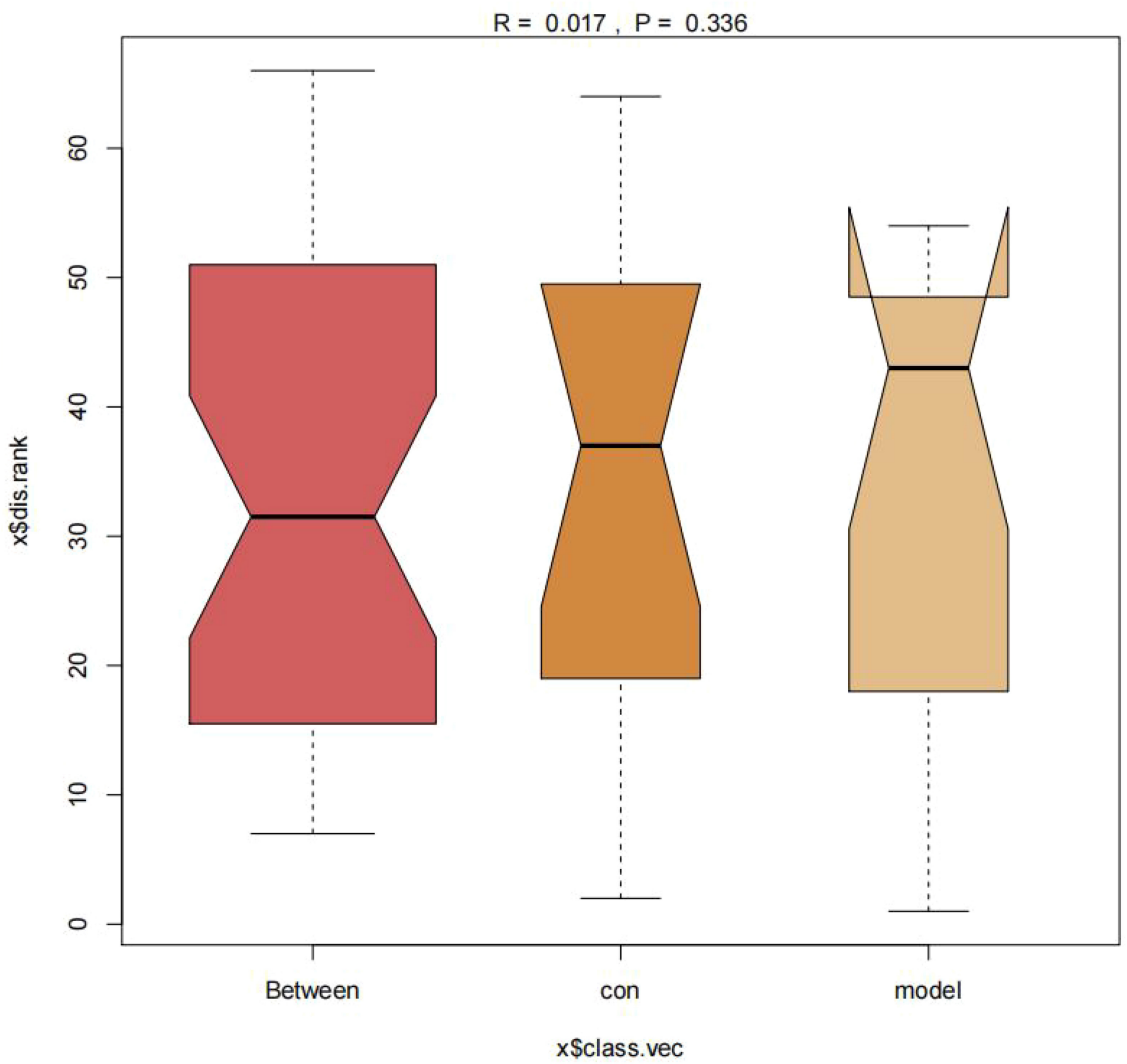
Between-group/within-group analysis of intestinal flora differences between model group and control group. The ordinate represents the rank of the distance between samples. The abscissa: “Between” represents the results between the two groups, and the other two represent the results within each group. The range of R value in the diagram is [–1, 1]. R > 0 means that the between-group difference is greater than the within-group difference, while R < 0 means that the between-group difference is less than the within-group difference. The confidence level of statistical analysis is represented by the *P*-value, where *P* < 0.05 indicates statistical significance. Con: control group, model: model group.

Anosim is a non-parametric test used to determine whether the difference between groups (either two or more groups) is significantly greater than the difference within groups, thereby determining if the grouping is meaningful. It does this through the analysis of the distance matrix between samples (default is based on Bray-Curtis distance analysis), resulting in an *R*-value. Based on the aforementioned Anosim analysis, ranks obtained from sorting the distance values between all pairwise samples (between groups labeled as between, within groups labeled as within) allow for the comparison of any two groups resulting in three groups of data, which can then be displayed in a box plot, as follows:

The study results showed that the difference in gut microbiota structure between the Model group and the Control group (inter-group difference) was greater than the difference within each group (*R* = 0.017 > 0) (Figure 7).

**FIGURE 8:**
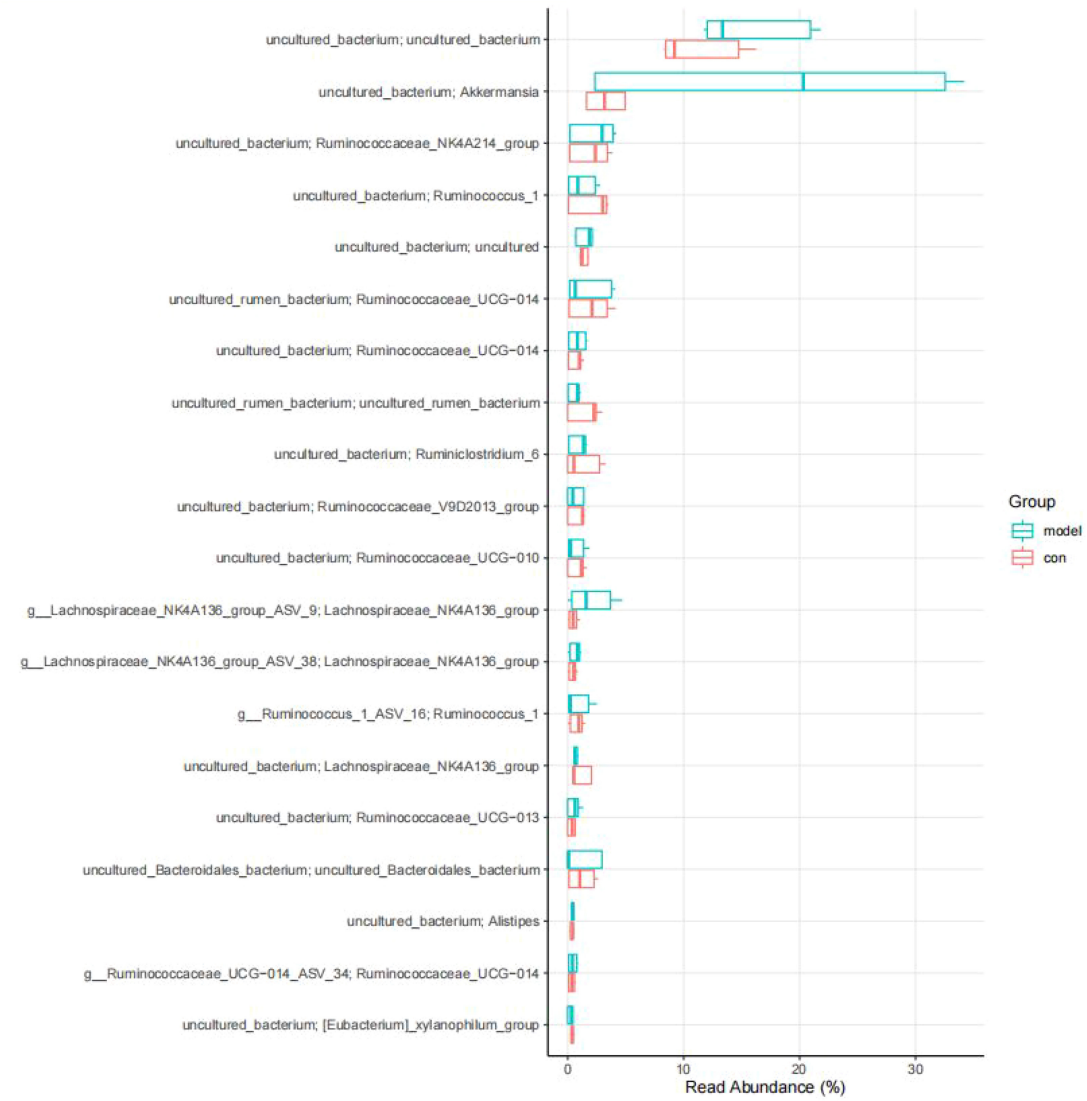
Difference in the abundance of TOP20 species between the model group and the control group. Each box plot in the figure shows, respectively, the minimum, first quartile, median, third quartile, and maximum of each α diversity index within the group samples; the vertical axis represents different species, and the horizontal axis represents the percentage of relative abundance.

Bacterial strains were ranked according to relative abundance from high to low, and the top 20 were chosen for the box plot below, which visually reflected the abundance difference of main bacterial strains between groups. The research results showed that at the level of bacterial classification, compared with the control group, the Model group had a higher relative abundance of Akkermansia and Lachnospiraceae; whereas the relative abundance of Ruminococcus and Ruminococcaceae was lower (Figure 8).

Our research found that Akkermansia and Lachnospiraceae are potential biomarkers for osteoarthritis. Our research team further studied their mechanisms and universality, and for the first time discovered the impact of prebiotics on the metabolic disorder of PTOA mouse joints, gut barrier and gut microbiota (Yilin et al., 2023).

## 5 Application of MAGMDA

**FIGURE 9:**
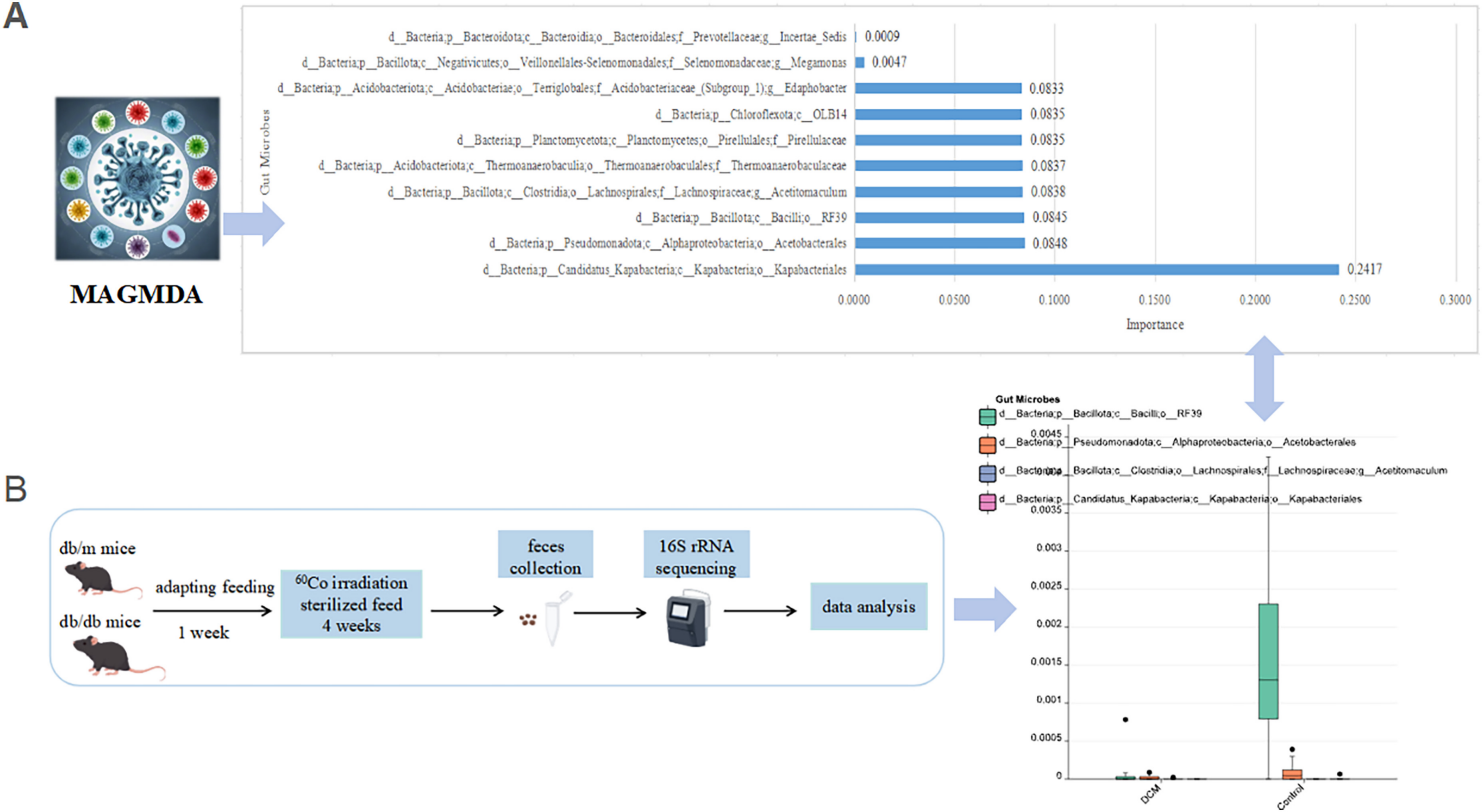
Application of MAGMDA. **(A)** MAGMDA-predicted gut microbial taxa associated with diabetic cardiomyopathy (DCM). The Feature Importance Plot shows the top 10 most relevant gut microbes. **(B)** Differential abundant gut microbes between db/db DCM model mice and control mice identified by 16S rRNA sequencing. Labels indicate: d__Bacteria; p__Candidatus_Kapabacteria; c__Kapabacteria; o__Kapabacteriales, d__Bacteria; p__Pseudo monadota; c__Alphaproteobacteria; o__Acetobacterales, d__Bacteria; p__Bacillota; c__Bacilli; o__RF39, d__Bacteria; p__Bacillota; c__Clostridia; o__Lachnospirales; f__Lachnospiraceae; g__ Acetitomaculum.

We employed MAGMDA to explore gut microbiota associated with diabetic cardiomyopathy (DCM). MAGMDA predicts associations at multiple taxonomic levels (genus: Acetitomaculum; family: Lachnospiraceae; phylum: Bacillota). Our 16S rRNA sequencing (V3-V4 region, primers 341F/806R) provides genus-level resolution at minimum. The identified taxa in animal experiments matched predicted levels: Lachnospiraceae at family level, Acetitomaculum at genus level (99% sequence similarity to reference database). For phylum-level predictions (Bacillota, Pseudomonadota), 16S data confirmed presence at relative abundances > 1% in model group. MAGMDA revealed a strong association between gut microbiota (e.g., d__Bacteria;p__Candidatus_Kapabacteria; c__Kapabacteria;o__Kapabacteriales, d__Bacteria;p__Pseudomo nadota;c__Alphaproteobacteria;o__Acetobacterales, d__Bacteria;p__Bacillota;c__Bacilli;o__RF39, d__Bacteria;p__Baci llota;c__Clostridia;o__Lachnospirales;f__Lachnospiraceae;g__ Acetitomaculum) and diabetic cardiomyopathy (Figure 9A). To validate the accuracy of the model’s predictions, we designed an animal experiment for verification. Six 8-week-old male db/m mice were used as the normal control group, and six 8-week-old male db/db mice were used as the model group. Both groups were fed an irradiated diet for 4 weeks to induce diabetic cardiomyopathy in the model group mice. All animals were housed in an environment with a temperature of (24 ± 2)°C, relative humidity of (50 ± 10) %, and a 12-h light/dark cycle, with free water and food. FBG ≥ 11.1 mmol/L, along with impaired cardiac function (as evidenced by echocardiography results), was considered indicative of successful model establishment. Fecal samples were aseptically collected from the mice, and DNA was extracted for 16S rRNA sequencing to compare the differences in gut microbiota composition between the two groups. The results of data analysis validated the gut microbiota identified by MAGMDA (Figure 9B).

## 6 Conclusion

In this study, we introduced MAGMDA, a graph convolutional network enhanced with a multi-layer attention mechanism, designed to predict associations between microorganisms and human diseases. By integrating known microbe-disease associations with similarity measures for both microbes and diseases into a heterogeneous network, MAGMDA effectively captures complex, nonlinear relationships that traditional computational methods often fail to model. The incorporation of attention mechanisms across graph convolution layers preserves multi-scale structural information, aims to mitigate the over-smoothing problem through multi-layer attention weighting; however, direct quantitative evidence of over-smoothing reduction (e.g., layer-wise embedding similarity analysis) is not provided in this study and remains an avenue for future investigation commonly encountered in deep graph-based models (Barrows et al., 2016; Zhou et al., 2017). Furthermore, the use of a weighted binary cross-entropy loss function mitigates the impact of class imbalance, improving the model’s sensitivity to positive associations despite data sparsity.

The model demonstrated robust predictive performance across two benchmark datasets, consistently outperforming existing methods in both AUC and AUPR. More importantly, its biological relevance was substantiated through case studies on asthma and type 2 diabetes, where the majority of top-ranked microbial candidates were corroborated by published literature. External validation using real-world clinical data and wet-lab experiments further provides preliminary supporting evidence for the model’s predictive capacity, though causal relationships remain to be established through interventional studies (e.g., fecal microbiota transplantation) to identify gut microbiota relevant to osteoarthritis and diabetic cardiomyopathy, reinforcing its translational potential in microbiome research.

By accurately prioritizing microbial candidates for experimental validation, MAGMDA offers a powerful computational tool to guide hypothesis generation and reduce the scope of costly and time-intensive biological experiments. Its application to gut microbiota-related diseases highlights its value in advancing our understanding of the gut–microbiome–host axis. Future work may extend this framework to incorporate multi-omics data, temporal dynamics, or cross-kingdom interactions, thereby enabling more comprehensive models of microbial contributions to human health and disease.

This study has several limitations that should be acknowledged. First, the benchmark datasets (HMDAD, Disbiome) are sparse (2–3% known associations) and may not fully represent the complexity of gut microbial communities. Second, the reported performance improvements (1–2% AUC/AUPR gains) are modest and, while statistically significant, may not translate to clinically meaningful improvements in predictive accuracy. Third, the external validation studies used small sample sizes (*n* = 6 per group for animal experiments) and clinical measurements that do not directly map to model predictions at the taxon level. Fourth, potential data leakage cannot be completely ruled out despite our precaution of recomputing similarity matrices within each CV fold. Fifth, the model was only tested on two disease types (asthma, type 2 diabetes) for case studies and two for external validation (osteoarthritis, diabetic cardiomyopathy); generalizability to other diseases remains unknown. Future work should address these limitations through larger-scale validation, multi-omics integration, and prospective cohort studies.

## Data Availability

The datasets presented in this study can be found in online repositories. The names of the repository/repositories and accession number(s) can be found in the article/supplementary material.

## Footnotes

1
http://www.cuilab.cn/hmdad

2
https://disbiome.ugent.be/home

3
https://meshb.nlm.nih.gov/

4
https://string-db.org/
